# Glucose Metabolism via the Entner-Doudoroff Pathway in *Campylobacter*: A Rare Trait that Enhances Survival and Promotes Biofilm Formation in Some Isolates

**DOI:** 10.3389/fmicb.2016.01877

**Published:** 2016-11-22

**Authors:** Christina S. Vegge, Melissa J. Jansen van Rensburg, Janus J. Rasmussen, Martin C. J. Maiden, Lea G. Johnsen, Morten Danielsen, Sheila MacIntyre, Hanne Ingmer, David J. Kelly

**Affiliations:** ^1^Department of Veterinary Disease Biology, Faculty of Health and Medical Sciences, University of CopenhagenCopenhagen, Denmark; ^2^Department of Zoology, University of OxfordOxford, UK; ^3^NIHR Health Protection Research Unit in Gastrointestinal InfectionsOxford, UK; ^4^MS-OmicsFrederiksberg, Denmark; ^5^School of Biological Sciences, University of ReadingReading, UK; ^6^Department of Molecular Biology and Biotechnology, The University of SheffieldSheffield, UK

**Keywords:** glycolysis, stationary-phase, hexose sugar, polysaccharide, capsule, PubMLST database

## Abstract

Isolates of the zoonotic pathogen *Campylobacter* are generally considered to be unable to metabolize glucose due to lack of key glycolytic enzymes. However, the Entner-Doudoroff (ED) pathway has been identified in *Campylobacter jejuni* subsp. *doylei* and a few *C. coli* isolates. A systematic search for ED pathway genes in a wide range of *Campylobacter* isolates and in the *C. jejuni/coli* PubMLST database revealed that 1.7% of >6,000 genomes encoded a complete ED pathway, including both *C. jejuni* and *C. coli* from diverse clinical, environmental and animal sources. In rich media, glucose significantly enhanced stationary phase survival of a set of ED-positive *C. coli* isolates. Unexpectedly, glucose massively promoted floating biofilm formation in some of these ED-positive isolates. Metabolic profiling by gas chromatography–mass spectrometry revealed distinct responses to glucose in a low biofilm strain (CV1257) compared to a high biofilm strain (B13117), consistent with preferential diversion of hexose-6-phosphate to polysaccharide in B13117. We conclude that while the ED pathway is rare amongst *Campylobacter* isolates causing human disease (the majority of which would be of agricultural origin), some glucose-utilizing isolates exhibit specific fitness advantages, including stationary-phase survival and biofilm production, highlighting key physiological benefits of this pathway in addition to energy conservation.

## Introduction

The zoonotic pathogen *Campylobacter* is the cause of human campylobacteriosis, the most frequently reported foodborne illness in Europe. The symptoms of campylobacteriosis are gastroenteritis with watery or bloody diarrhea, and the disease is in the majority of cases self-limiting (Butzler, 2004). The most prominent *Campylobacter* species causing disease in humans are *C. jejuni* and *C. coli*, which are most frequently associated with the consumption or handling of contaminated animal products, especially poultry, but also with animal or environmental contact (Kaakoush et al., 2015). *C. jejuni* is divided into two subspecies: *C. jejuni* subsp. *jejuni*, and *C. jejuni* subsp. *doylei*, which are distinguished by the inability of the latter to reduce nitrate to nitrite (Miller et al., 2007).

**FIGURE 1:**
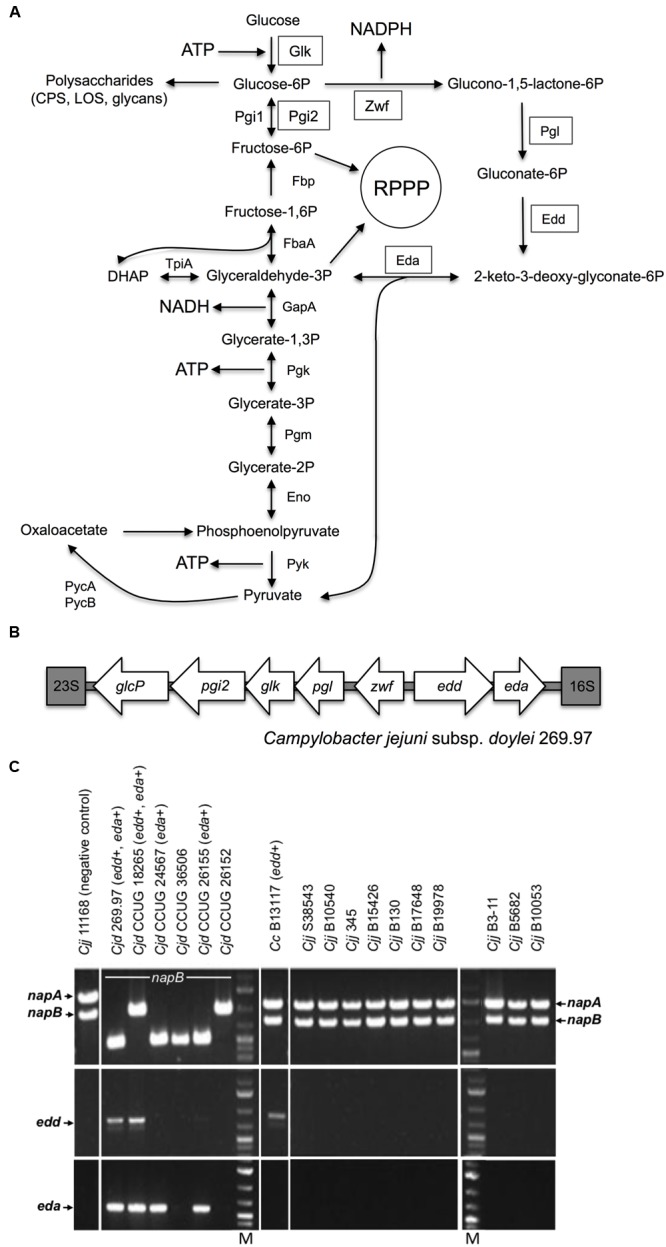
**The Entner-Doudoroff pathway in *Campylobacter* isolates. (A)** Scheme of the ED pathway to illustrate how the reactions effectively bypass the lack of phosphofructokinase, which prevents glycolysis by the EMP pathway in *Campylobacter*, but can also allow cycling of triose-phosphate back to hexose phosphate. The key enzymes 6-phosphogluconate dehydratase (Edd) and 2-keto-3-deoxy-6-phosphogluconate aldolase (Eda) catalyze the dehydration of *phosphate back to hexose gluconate-6P to 2-keto-3-deoxy-gluconate-6P and the further aldol cleavage to pyruvate and glyceraldehyde-3P, respectively. Inaddition, the ED pathway requires a glucokinase (Glk), a glucose-6-phosphate dehydrogenase (Zwf) and a 6-phosphogluconolactonase (Pgl) for conversion ofglucose to gluconate-6P, the substrate of Edd. Two phosphoglucose isomerases (Pgi1 and Pgi2) catalyze the same reaction. ED pathway specific enzymes are boxed; Pgi2 is encoded at the *glc* locus (Vorwerk et al., 2015) and may be required to feed fructose-6-phosphate into the reductive pentose phosphate pathway (RPPP). **(B)** Organization of the *glc* locus encoding the ED pathway in *Campylobacter jejuni* subsp. *doylei* 269.97. The locus is situated between the 16S and 23S ribosomal rRNA genes and contains the genes *pgi2, glk, pgl, zwf, edd* and *eda* in addition to the glucose transporter *glcP*. **(C)** Screen for *napA, napB, edd*, and *eda* genesin *Campylobacter* isolates. Top panels show PCR fragments of the nitrate reductase genes *napA* (internal fragment) and *napB* (flanking fragment) used to discriminate *C. jejuni* subsp. *doylei* (*Cjd*) from *C. jejuni* subsp. *jejuni* (*Cjj*) and *C. coli (Cc)*. *Cjj* and *Cc* encode both *napA* and *napB* and thus the ability to reduce nitrate. In contrast, *Cjd* has deletions in *napA* and sometimes also *napB*, thus leaving this subsp. unable to reduce nitrate (Miller et al., 2007). None of the *Cjd* isolates showedamplicons with the *napA* internal primers. The *napB* diversity of *Cjd* is illustrated by the variable fragment lengths amplified with the *napB* flanking primers, as described by Miller et al. (2007). Middle panels show amplified ca. 810 bp fragments of the *edd* gene from *Cjd* 269.97, *Cjd* CCUG 18265 and *Cc* B13117. Bottompanels show amplified ca. 600 bp fragments of the *eda* gene from *Cjd* 269.97, *Cjd* CCUG 18265 *Cjd* CCUG 24567, and *Cjd* CCUG 26155. Lanes M contain molecular size markers.*

Campylobacters are fastidious, microaerophilic, host-adapted organisms with a metabolic capacity highly tuned to their biological niche. For *C. jejuni*, the most widely used carbon sources are primarily the amino acids aspartate, glutamate, serine, and proline (Velayudhan et al., 2004; Guccione et al., 2008; Hofreuter, 2014) as well as certain peptides and organic acids such as lactate, pyruvate and intermediates of the citric acid cycle (Wright et al., 2009; Thomas et al., 2011; Hofreuter, 2014). For many years, the general consensus has been that *Campylobacter* isolates are unable to catabolise various sugars, especially glucose, due to the specific lack of glucokinase (Glk) and phosphofructokinase (PfkA) of the classical Embden-Meyerhof-Parnas (EMP) glycolysis pathway, while the presence of the remaining EMP enzymes (**Figure 1A**) allows the anabolic generation of hexose phosphate via the reverse reactions of gluconeogenesis (Parkhill et al., 2000; Velayudhan and Kelly, 2002). However, catabolism of L-fucose, a hexose sugar, was recently observed in some *C. jejuni* isolates, which overturned this view (Muraoka and Zhang, 2011; Stahl et al., 2011). L-fucose is abundantly present in the mucosal layer of intestinal epithelial cells, originating from fucosylated mucin glycoproteins, and is taken up via the fucose permease FucP (Stahl et al., 2011). The *fucP* gene was found in 30.3% of 710 *C. jejuni* isolates by de Haan et al. (2012) and 57.9% of 266 *C. jejuni* isolates by Zautner et al. (2012), indicating that the ability to utilize L-fucose is quite common but not universal. The genes for L-fucose catabolism are encoded on a genomic island (Stahl et al., 2011) and in *C. coli* isolates, there is evidence that these genes have been introgressed from *C. jejuni* (Sheppard et al., 2013). Significantly, *C. jejuni* mutants lacking the FucP permease displayed a competitive disadvantage in colonization of both chickens and piglets (Stahl et al., 2011). The mechanism of L-fucose catabolism in *Campylobacter* has yet to be fully characterized enzymologically, but seems to proceed by a set of reactions involving non-phosphorylated intermediates, likely forming 1 mol pyruvate and 1 mol lactate per mol L-fucose (Stahl et al., 2011).

Alternative routes to the EMP pathway for the catabolism of glucose include the oxidative pentose phosphate (PP) and the Entner-Doudoroff (ED) pathways. The seemingly universal lack of gluconate-6-phosphate dehydrogenase in campylobacters means the remaining PP enzymes form a purely anabolic pathway. However, the ED pathway genes and a possible glucose transporter gene were first observed in the sequence of *C. jejuni* subsp. *doylei* 269.97 (**Figure 1B**) and proposed as a theoretical way of glucose utilization in this isolate by Miller (2008). Importantly, whereas the EMP pathway produces 2 mol ATP and 2 mol NADH per mol glucose, the ED pathway produces 1 mol each of ATP, NADH, and NADPH per mol glucose (Flamholz et al., 2013). Mechanistically, the ED pathway is somewhat similar to the EMP pathway in that it involves initial activation of the C6 sugar by phosphorylation and a subsequent aldol cleavage to give two C3 intermediates (Conway, 1992), but unlike the EMP pathway the ED aldolase reaction yields one mol of triose phosphate and one mol of pyruvate directly. The key enzymes are 6-phosphogluconate dehydratase (Edd) and 2-keto-3-deoxy-6-phosphogluconate aldolase (Eda) (Conway, 1992; Flamholz et al., 2013). Edd catalyzes the dehydration of gluconate-6P to 2-keto-3-deoxy-gluconate-6P, while Eda catalyzes the aldol cleavage of this compound to pyruvate and glyceraldehyde-3P. In addition, the ED pathway requires a glucokinase (Glk), a glucose-6-phosphate dehydrogenase (Zwf) and a 6-phosphogluconolactonase (Pgl) for conversion of glucose to gluconate-6P, the substrate of Edd (**Figure 1A**).

There are a number of variations of the ED pathway in different groups of bacteria (Conway, 1992). For example, in *Zymomonas mobilis* (a common cause of spoilage of fermented beverages) the pathway is constitutive, effectively linear and is the sole mechanism of converting glucose to pyruvate. In enteric bacteria like *Escherichia coli*, the ED pathway enzymes are inducible and serve primarily in gluconate catabolism, with glucose itself being catabolized preferentially by EMP-mediated glycolysis (Conway, 1992). Gluconate is present in the intestine; interestingly, although campylobacters cannot catabolise gluconate (Pajaniappan et al., 2008; Vorwerk et al., 2015), *C. jejuni* has been shown to use it as an electron donor for respiration via a periplasmic gluconate dehydrogenase (Pajaniappan et al., 2008). A third “cyclic” ED pathway variation is found in *Pseudomonas* spp. that, like campylobacters, lack 6-phosphofructokinase. Here, there is evidence from labeling studies (Nikel et al., 2015) that a portion of the triose-phosphate formed by the Eda aldolase reaction is cycled back to hexose phosphate via the gluconeogenic reactions of the EMP pathway (see **Figure 1A**); this has recently been dubbed the “EDEMP” pathway (Nikel et al., 2015). From genomic studies it seems that the ED pathway (and its variants) is much more common in facultative and obligate aerobes, while anaerobes rely on the EMP pathway (Flamholz et al., 2013). One attractive hypothesis is that both triose-phosphate and pyruvate can be produced with far fewer enzymes by the ED pathway compared to the EMP pathway, which can be viewed as a trade-off with the reduced ATP yield (Flamholz et al., 2013). This may be of particular importance for glucose utilization in small genome, host adapted pathogens.

Recently, in an elegant and detailed study using mutagenesis, ^13^C-isotopolog analysis and enzyme studies, Vorwerk et al. (2015) discovered that a functional ED pathway exists in certain *C. coli* isolates, which enables this pathogen to utilize glucose as a growth substrate. Moreover, the pathway (like that for L-fucose) was found to be encoded on a genomic island or ‘plasticity region,’ designated the *glc* locus, that could be transferred between ED-positive and ED-negative isolates by natural transformation, suggesting acquisition of the *glc*-enoded ED pathway could contribute to the expansion of metabolic diversity in campylobacters (Vorwerk et al., 2015). However, knowledge of the actual distribution of the ED pathway genes in isolates from a range of different sources is lacking and it is thus not clear what the role or importance of glucose utilization is in the context of the known genomic diversity of campylobacters.

In this study we show by systematic genomic analyses that the ED pathway genes are present in only 1.7% of >6,000 genomes encompassing a diverse range of isolates of both of the major pathogenic species of *Campylobacter* (*C. jejuni* and *C. coli*). However, we demonstrate that the ED pathway provides a significant benefit for *C. coli* in prolonged growth experiments in rich media, where ED-positive isolates display significantly extended stationary-phase survival in the presence of glucose. Interestingly, metabolic profiling revealed that individual ED-positive isolates can respond very differently to glucose, correlating with glucose-stimulated formation of a pellicle or floating biofilm in some isolates. We conclude that although the ability to utilize glucose is generally uncommon in *Campylobacter*, it is found in a more diverse range of isolates than previously suspected and we provide evidence that the ED pathway, in addition to a purely catabolic role, also confers distinct physiological advantages in survival and biofilm formation in some isolates.

## Materials and Methods

### Bacterial Isolates and Growth Conditions

The origins and details of the *Campylobacter* isolates used in this study are given in Supplementary Table S1. Isolates were routinely cultivated on Blood Agar Base No. 2 (Oxoid) supplemented with 5% (v/v) bovine blood and incubated at 37°C under microaerobic conditions (6% v/v CO_2_; 6% v/v O_2_; 3.4% v/v H_2_, and 80.6% v/v N_2_). Growth experiments were carried out with and without 100 mM glucose supplementation in Tryptic Soy Broth (TSB) without Dextrose (Becton Dickinson and Co.) or in modified MCLMAN minimal media (Alazzam et al., 2011) without lactate and with 2 mM aspartate. Viable cell counts were determined by plating serial dilutions in phosphate buffered saline on Blood Agar Base No. 2 (Oxoid) and incubating under the conditions described above.

### PCR Screen for ED Pathway Genes

*Campylobacter* isolates (Supplementary Table S1) were screened for the ED pathway genes by PCR using oligonucleotides for *edd* (5′-ATAAATTGGGATGATTTTG and 5′-TCTAAACCCTGCAAAAAGCTC giving a ca 810 bp fragment) and *eda* (5′-GGAGAAAAAATGCAAACAA and 5′-TCTAAACCCTGCAAAAAGCTC giving a ca 600 bp fragment). Oligonucleotides for *napA* and *napB* (periplasmic nitrate reductase) were used in multiplex PCR reactions to discriminate *C. jejuni* subsp. *doylei* from *C. jejuni* subsp. *jejuni* and *C. coli* using the *nap* mpx2 primer set as described by Miller et al. (2007), which consists of internal *napA* primers and *napB* flanking primers. Chromosomal DNA was extracted with the DNeasy Blood and Tissue kit (Qiagen) and the DreamTaq Green DNA polymerase (Thermo Fisher) was applied for the PCR amplifications.

### Whole Genome Sequencing and Assembly

Genomic DNA was extracted from bacterial cultures of CCUG 18265, CCUG 24567, CCUG 26155, B13117, and CV1257 using the Wizard Genomic DNA Purification Kit (Promega, Southampton, UK). Whole-genome sequencing (WGS) was carried out at the Wellcome Trust Sanger Institute, UK. Illumina multiplex libraries were generated by acoustic shearing, after which up to 96 libraries were pooled in equimolar ratios and sequenced on a single flow cell lane on the Illumina HiSeq platform, producing 100 bp paired-end reads, as previously described (Cody et al., 2013). The short-read data were submitted to an automated pipeline (Jolley and Maiden, 2010; Bratcher et al., 2014), which integrates VELVET version 1.2.01 (Zerbino and Birney, 2008) and VELVETOPTIMISER version 2.2.0 (Zerbino, 2010) for *de novo* assembly. Draft genomes were then uploaded to the *C. jejuni*/*coli* PubMLST database (Supplementary Table S2).

### Genome Annotation and *In silico* Identification of ED-Positive Isolates

Loci corresponding to *glcP* (CAMP2017), *pgi2* (CAMP2018), *glk* (CAMP2019), *pgl* (CAMP2020), *zwf* (CAMP2021), *edd* (CAMP2022), and *eda* (CAMP2023) were added to the *C. jejuni*/*coli* PubMLST database (PubMLST locus names shown in parentheses), using sequences from *C. jejuni* subsp. *doylei* 269.97 (GenBank accession number NC_009707) to seed the database. The *glc* loci, in addition to MLST, rMLST, and *C. jejuni*/*coli* core genome scheme loci, were annotated in the study genomes using the Bacterial Isolate Genome Sequence Database (BIGSDB) ‘autotagger’ functionality implemented in PubMLST (Jolley and Maiden, 2010). The autotagger uses BLAST to search a genome for sequences similar to loci defined in the database. For sequences with ≥98% identity to existing alleles, the autotagger defined the position of the locus and assigned a unique allele number in order of discovery (Jolley and Maiden, 2010). Sequences with <98% identity to existing alleles were curated manually. The same approach was used to annotate components of the *glc* locus in publicly available genomes present in the *C. jejuni*/*coli* PubMLST database. A summary of the allelic data for MLST and *glc* loci from all ED-positive isolates was generated using the BIGSDB data export plugin. ED types were generated as for MLST sequence types: unique combinations of alleles across the *glc* loci, taken in gene order from *glcP* to *eda*, were assigned arbitrary numbers in order of discovery.

### Genomic Analyses

Genomic analyses were carried out using the hierarchical gene-by-gene approach implemented in BIGSDB, which allows users to compare isolates at varying numbers of loci, depending on the level of resolution required (Jolley and Maiden, 2010; Maiden et al., 2013). Species assignments of all ED-positive isolates, and *C. coli* clade membership, were confirmed using rMLST (Jolley et al., 2012; Jansen van Rensburg et al., 2016). A maximum likelihood tree based on concatenated nucleotide sequences of the *glc* genes was reconstructed in MEGA version 6.06 (Tamura et al., 2013) using the General Time Reversible model with gamma-distributed rates with 500 bootstrap replicates. The resulting phylogenetic tree was annotated online using the Interactive Tree of Life version 3 (Letunic and Bork, 2016). Relationships among ED-positive isolates were established based on wgMLST analyses carried out using the GENOME COMPARATOR module implemented in the *C. jejuni*/*coli* PubMLST database (Jolley and Maiden, 2010). Following the exclusion of genomes in which ≥1% of the 1,343 *C. jejuni*/*coli* core genome scheme loci were incomplete (i.e., at the ends of contigs), relationships among *C. jejuni* and *C. coli* isolates were evaluated using wgMLST. Isolates were compared to the reference genomes of *C. jejuni* NCTC11168 (GenBank accession number AL111168) (Parkhill et al., 2000; Gundogdu et al., 2007) or *C. coli* 15-537360 (CP006702) (Pearson et al., 2013), using the default GENOME COMPARATOR settings. Distance matrices generated by GENOME COMPARATOR were visualized as networks using the NeighborNet algorithm (Bryant and Moulton, 2004) in SplitsTree version 4.13.1 (Huson and Bryant, 2006). Further analyses were carried out to compare the isolates B13117 and CV1257. The genome sequences of these isolates were annotated using Prokka version 1.0 (Seemann, 2014). The number of shared and unique coding sequences was estimated with Roary version 3.6.0 (Page et al., 2015) using the default settings. The capsule gene regions of these isolates were compared using ACT (Carver et al., 2005).

### Phenotype Microarray Assays

BIOLOG^TM^ phenotype microarrays were set up according to the manufacturer’s description for *C. jejuni*. Briefly, individual isolates were cultivated overnight on Blood Agar Base No. 2 supplemented with 5% v/v bovine blood at 37°C in a microaerobic atmosphere. Cells were harvested from plates with 1.2x IF-0a solution (Biolog, Inc.), gently resuspended and adjusted to an optical density (600 nm) of 0.8. For each strain, 8 ml cell suspension was mixed with 12 ml 1.2x IF-0a (Biolog, Inc.), 0.24 ml Dye mix D (Biolog, Inc.), 6 mg ml^-1^ BSA, 1.26 mg ml^-1^ NaHCO_3_, and 1.76 ml water. This mixture (0.1 ml) was added to the wells of PM1 phenotype microarray plates (Biolog), and the initial absorbance at 590 nm was read in an ELISA plate reader. Plates were incubated at 37°C in a microaerobic atmosphere and the absorbance read again following 24 and 48 h of incubation.

### Biomass Dry-Weight Determination

Following 7 days of incubation at 37°C in a microaerobic atmosphere, *C. coli* cultures in glucose-free TSB with and without supplementation with 100 mM glucose were homogenized carefully by pipetting. Biomass was harvested from 5 ml culture by centrifugation and the pellets incubated overnight at 50°C for evaporation of water content. The dry weight of biomass was determined as the mean of four replicates.

### Metabolite Analysis by Gas Chromatography–Mass Spectrometry

*Campylobacter coli* B13117 and CV1257 were cultivated at 37°C under microaerobic conditions in TSB with and without supplementation with 100 mM glucose. Three independent cultures for each strain and condition were grown; following 24 h of incubation, cells were harvested from 5 ml culture by centrifugation for 1 min at 8,000 × *g* at 0°C. The supernatants were filter sterilized (0.2 μm pore size) and stored at -20°C until analysis. A procedure based on the methyl chloroformate derivatisation protocol described by Smart et al. (2010) was used for the analysis of mixtures containing known metabolites with and without complexation. All samples were analyzed in a randomized order. The system was controlled by ChemStation (Agilent technologies). Raw data was converted to netCDF format using Chemstation (Agilent), before the data was imported into Matlab R2014b (Mathworks, Inc., Natick, MA, USA) and processed using PARAFAC2 (Bro et al., 1999; Kiers et al., 1999) to obtain relative concentrations for each peak. PARAFAC2 was applied using in-house algorithms.

## Results

### Carriage of the ED Pathway Genes Does Not Correlate with Ability to Grow in Blood

The key ED pathway genes were originally identified in the genome sequence of the human blood isolate *C. jejuni* subsp. *doylei* 269.97 (Miller, 2008). Since *C. jejuni* subsp. *doylei* is overrepresented in human cases of *Campylobacter* bacteremia (Lastovica, 2006; Parker et al., 2007) and given the millimolar concentrations of glucose in blood, we initially wanted to test the hypothesis that the ED pathway might be specifically associated with the growth or survival of *Campylobacter* found in blood. Therefore, a range of *Campylobacter* isolates was screened for the ED pathway using PCR amplification of the key genes (**Figure 1C**). This screen included five clinical isolates of *C. jejuni* subsp. *doylei* (CCUG 18265, CCUG 24567, CCUG 26152, CCUG 26155, CCUG 36506), a *C. coli* bacteremia isolate (B13117), an in-house *C. coli* isolate of unknown origin (CV1257) and 10 *C. jejuni* subsp. *jejuni* bacteremia isolates (S38543, B10540, B345, B15426, B130, B17648, B19978, B3-11, B5682, B10053) (see Supplementary Table S1 for details). The screen revealed the presence of 6-phosphogluconate dehydratase (*edd*) and/or aldolase (*eda*) genes, encoding the key ED pathway enzymes, in three out of five clinical *C. jejuni* subsp. *doylei* isolates (CCUG24567, CCUG18265, CCUG26155), both *C. coli* isolates (B13117, CV1257), but not in any of the examined *C. jejuni* subsp. *jejuni* bacteremia isolates (**Figure 1C** and results not shown). These findings therefore do not support an association between blood culture isolates and the key genes of the *glc* locus. Analysis of WGS data for CCUG 24567, CCUG 18265, CCUG 26155, B13117, and CV1257 (Supplementary Table S2) confirmed that all isolates carried the complete *glc* locus.

### *In silico* Identification of ED-Positive *Campylobacter* Isolates

The availability of assembled WGS data in the *C. jejuni*/*coli* PubMLST database facilitated large-scale *in silico* searches for additional ED-positive isolates. We found that 113 out of 6,184 isolates with genomic data contained one or more components of the *glc* locus. The *glc* locus did not assemble into a single contig in six isolates, all of which corresponded to draft genomes. Breaks occurred within the genes of interest, likely due to misassembly or lack of coverage; these isolates were excluded from further analyses. The complete *glc* locus was present on a single contig in the remaining 107 isolates (1.7% of the total) (Supplementary Table S2), which were collected in the UK (*n* = 76), Finland (*n* = 30), and the USA (*n* = 1).

Based on ribosomal multilocus sequence typing (rMLST) (Jolley et al., 2012), 70 and 37 isolates corresponded to *C. jejuni* and *C. coli*, respectively. At the population level, *C. coli* isolates segregate into three groups known as clades 1, 2, and 3 (Sheppard et al., 2008; Sheppard et al., 2010); the ED-positive *C. coli* isolates were all assigned to clade 1, except OXC7653, which belonged to clade 3. With respect to sources of isolation, *C. jejuni* isolates were predominantly from rats (51.4%) and wild birds (42.9%). In contrast, human disease samples accounted for 43.2% of *C. coli* isolates, while the remainder were from a diverse range of sources, including animals, food, and the environment (**Table 1**).

**Table 1:** Sources of ED-positive isolates from the *Campylobacter jejuni*/*coli* PubMLST database.

Source	*n* (%)	
	*C. jejuni*	*C. coli*
Chicken	0 (0)	1 (2.7)
Environmental waters	0 (0)	4 (10.8)
Farm environment	2 (2.9)	1 (2.7)
Human disease	0 (0)	16 (43.2)
Other food	0 (0)	2 (5.4)
Pig	0 (0)	5 (13.5)
Rat	36 (51.4)	3 (8.1)
Soil	0 (0)	3 (8.1)
Unknown	2 (2.9)	2 (5.4)
Wild bird	30 (42.9)	0 (0)

### Allelic Diversity of the *glc* Locus

**FIGURE 2:**
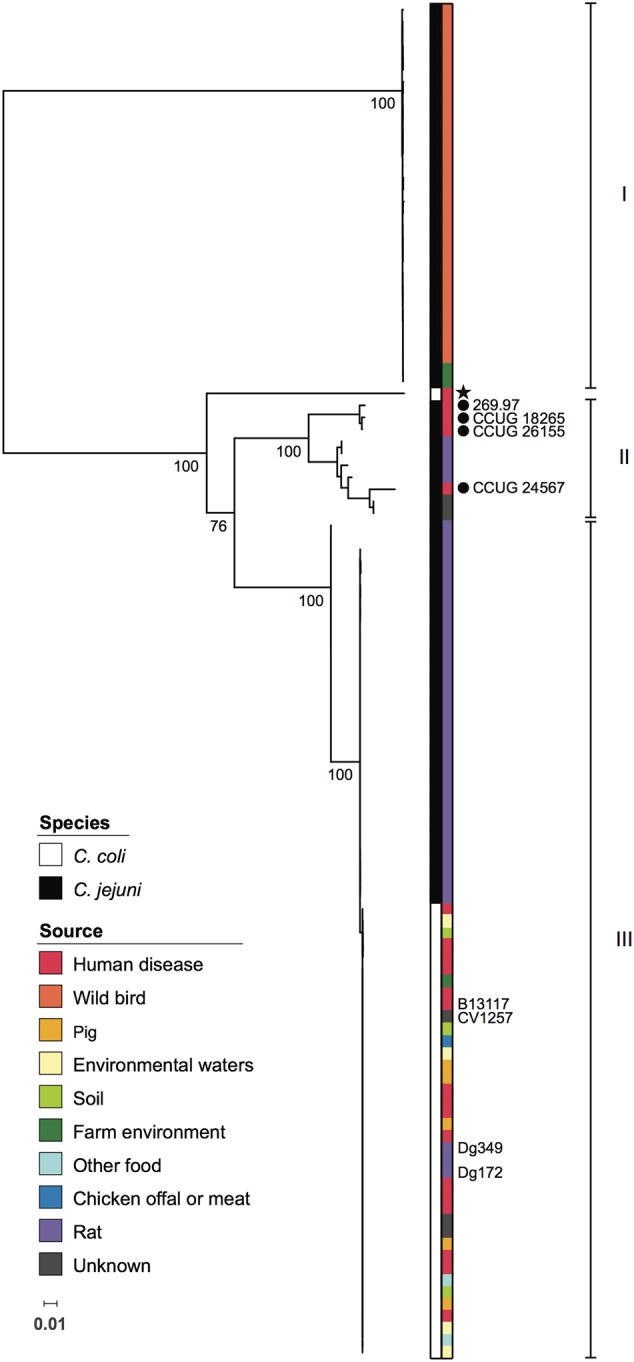
**Phylogenetic relationships among *Campylobacter glc* loci.** Maximum likelihood tree based on concatenated nucleotide sequences of genes comprising the *glc* locus, encoding the Entner-Doudoroff pathway, from 113 *Campylobacter* isolates. Colored strips adjacent to the phylogeny indicate species and source as shown in the inset legends. Known *C. jejuni* subsp. *doylei* isolates are indicated with filled circles. All *C. coli* isolates were assigned to *C. coli* clade 1, with the exception of a single clade 3 isolate, which is marked with a star. Isolates included in experiments carried out in this study are labeled. Roman numerals indicate groups of *glc* sequences referred to in the text. For major nodes, bootstrap values generated from 500 replicates are shown as percentages. The scale bar represents the number of nucleotide substitutions per site.

The seven genes comprising the *glc* locus were annotated as described in Experimental Procedures, using the autotagger functionality implemented in PubMLST (Jolley and Maiden, 2010). Unique alleles identified for each gene were assigned arbitrary numbers in order of discovery. Between 12 (*eda*) and 22 (*pgi2*) alleles per *glc* locus gene were present among *C. jejuni* subsp. *doylei* 269.97, the five ED-positive isolates sequenced for this study, and those from the *C. jejuni*/*coli* PubMLST database (**Table 2**). Gene lengths ranged from 624 bp (*eda*) to 1803 bp (*edd*), and allele lengths were variable for all loci except *edd, eda*, and *pgl* (**Table 2**). Unique combinations of *glc* locus alleles, taken in gene order from *glcP* to *eda*, were summarized as ED types, which were assigned arbitrary numbers in order of discovery. Overall, 46 ED types were identified, all of which were species-specific: 36 were present in *C. jejuni* and 10 in *C. coli*. *C. jejuni* isolates were evenly distributed across ED types, with the exception of 18 closely related rat isolates that had been isolated from four different farms between 2011 and 2012 and which all carried ED type 2 (ED allelic profile 3-4-4-5-4-5-5). Interestingly, these findings with *C. jejuni* contrasted with a more limited allelic diversity observed for *C. coli* isolates, amongst which ED type 1 (3-5-7-9-5-9-9) was predominant, accounting for 27/37 (73%) of PubMLST isolates and CV1257. Phylogenetic analysis of concatenated *glc* gene sequences indicated that ED types segregated into three groups, with *C. jejuni* ED types occurring in all three (**Figure 2**). There was little diversity among *C. jejuni* sequences from groups I and III, which primarily corresponded to isolates obtained from wild birds or rats, respectively. Sequences belonging to group II were more diverse and included the known *C. jejuni* subsp. *doylei* isolates. With the exception of clade 3 isolate OXC7653, *C. coli* sequences were only found in group III and were highly homogeneous (**Figure 2**).

**Table 2:** Allelic diversity of components of the *glc* locus among 113 ED-positive *Campylobacter* isolates.

Gene	Number of alleles	Length of seed sequence	Range of allele lengths
*glcP*	20	1212	1200–1215
*pgi2*	22	1644	1644–1688
*glk*	19	999	999–1014
*pgl*	15	681	681
*zwf*	13	1401	1398–1401
*edd*	20	1803	1803
*eda*	12	624	624

### Genomic Analyses of ED-Positive Isolates

**FIGURE 3:**
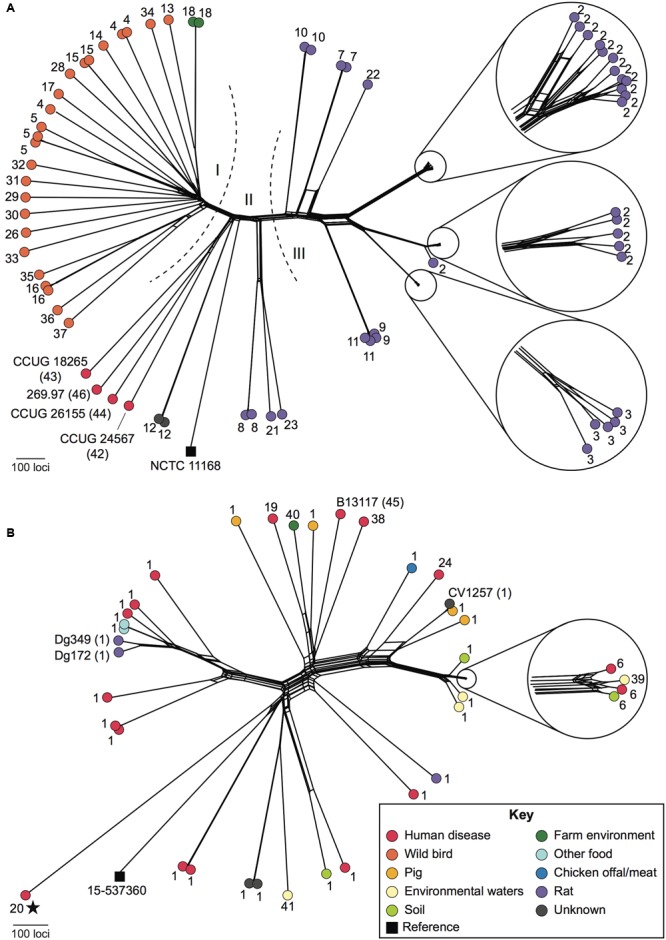
**Relationships among ED-positive *Campylobacter* isolates.** NeighborNet graphs were generated based on whole-genome multilocus sequence typing (wgMLST) comparisons of 68 *C. jejuni* **(A)** and 38 *C. coli* **(B)** isolates, using NCTC11168 and 15-537360 (black squares), respectively, as reference genomes. ED allele types are shown as numbers adjacent to each isolate. Isolates included in experiments carried out in this study are labeled in full with the ED type in parentheses. Dashed lines and Roman numerals indicate groups of isolates with related ED types referred to in the text. The color of the filled circles indicates the source of each isolate as shown in the key (inset). All *C. coli* were assigned to *C. coli* clade 1 except for a single clade 3 isolate, which is marked with a star.

Relationships among ED-positive isolates were examined using whole-genome multilocus sequence typing (wgMLST). Seven isolates were excluded from these analyses as 1–16.5% of loci belonging to the *C. jejuni*/*coli* core genome scheme were incomplete (i.e., at ends of contigs). With respect to *C. jejuni*, wild bird, rat, and known *C. jejuni* subsp. *doylei* isolates largely occupied distinct parts of the network that correlated with membership of the three ED nucleotide sequence groups (**Figures 2** and **3**). Members of these groups were genetically diverse and were separated by an average of 1,294 loci, with the exception of small clusters of closely related isolates, particularly among those obtained from farm-associated rats (**Figure 3A**). Isolates belonging to these clusters were typically separated by fewer than 100 loci and the majority carried ED type 2. Although *C. coli* isolates were also genetically diverse, they were separated by shorter distances, averaging 973 loci (**Figure 3B**). While the majority carried ED type 1, these isolates did not group by source or ED type.

### Glucose Supports Growth of *Campylobacter* Isolates Harboring the ED Pathway

The presence of the ED pathway genes within the subset of *Campylobacter* isolates studied here indicates that these isolates should be able to utilize glucose. To confirm this, a Biolog^TM^ phenotypic microarray was initially used to examine the potential metabolism of various carbohydrates by the ED-positive isolates *C. coli* B13117, *C. coli* CV1257 and *C. jejuni* subsp. *doylei* 269.97 in comparison to the widely used lab strain *C. jejuni* NCTC11168, which does not carry the ED pathway. With the Biolog^TM^ system, substrate uptake and metabolism is detected as stimulation of bacterial respiration and quantitated colorimetrically via tetrazolium dye reduction (Bochner et al., 2001). This revealed a correlation between the presence of the ED pathway and metabolism of glucose, as glucose was seen to stimulate respiration of the ED-positive *C. jejuni* subsp. *doylei* 269.97, *C. coli* B13117 and *C. coli* CV1257, while no signal was observed for *C. jejuni* NCTC11168. In contrast, L-fucose was seen to stimulate respiration of all four isolates regardless of the presence of the ED pathway (Supplementary Figure S1).

**FIGURE 4:**
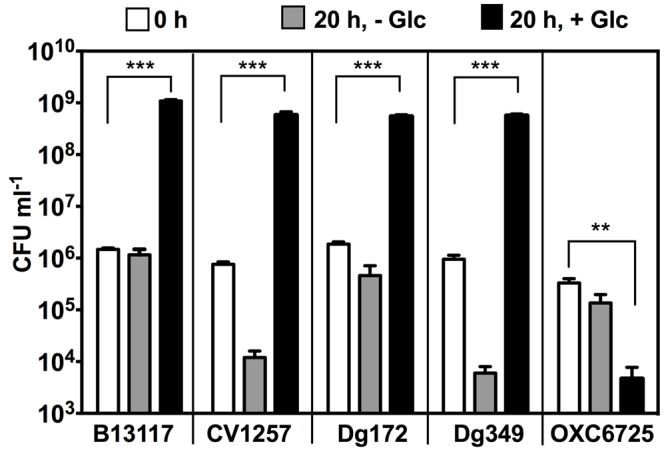
**Glucose stimulates growth of ED-positive but not ED-negative *C. coli* isolates.** Four ED-positive *C. coli* isolates (B13117, CV1257, Dg172, and Dg349) and an ED-negative control *C. coli* strain (OXC6725) were inoculated into modified MCLMAN minimal media either without or with glucose supplementation. Cultures were incubated for 20 h at 37°C under microaerobic conditions and viable cell numbers measured by plate counts. White bars, colony forming units (CFU) ml^-1^ of inoculum at time 0. Light gray bars, final CFU ml^-1^ in modified MCLMAN without glucose. Black bars, final CFU ml^-1^ in modified MCLMAN supplemented with 100 mM glucose. The bars show the mean and the error bars show the standard deviations of three independent cultures. Statistical significance is indicated by (^∗∗^*p* < 0.01) or (^∗∗∗^*p* < 0.001) as determined by Student’s *t*-test.

To investigate if glucose is able to support growth of *C. coli* isolates carrying the ED pathway, the growth of four ED-positive *C. coli* isolates representing three different sequence types (B13117, CV1257, Dg172, and Dg349) was examined in comparison to an ED-negative strain (OXC6725; PubMLST id 18282). Of these isolates, CV1257, Dg172, and Dg349 all had ED type 1 (the same as in the isolates used by Vorwerk et al., 2015), while B13117 carried ED type 45, which only differed by a single synonymous substitution in *pgi2* (471: T- > C). The isolates were grown in minimal media (MCLMAN) without and with glucose supplementation as the main carbon source; a low concentration of L-aspartate (2 mM) was included as a nitrogen source as this was found to greatly aid growth. Following overnight incubation at 37°C under microaerobic conditions, the viable cell count of each of the ED-positive isolates had increased significantly with glucose (by ∼3 logs), while no such increase in cell density was seen for either the ED-negative strain or any of the cultures without glucose supplementation (**Figure 4**). These data show a clear correlation between possession of the ED pathway in these *C. coli* isolates and their growth on glucose.

### Extended Stationary Phase Survival and Strain-Specific Biofilm Production with Glucose

In order to examine the physiological impact of glucose metabolism by *C. coli*, the growth of ED-positive isolates was further examined in the rich medium tryptic soy broth (TSB) without and with glucose supplementation. Here, a range of carbon sources (mainly amino-acids and peptides), are available in addition to the added glucose. At 37°C in a microaerobic atmosphere, the exponential growth rates of B13117 and CV1257 were seen to be unaffected by the availability of glucose (Supplementary Figure S2). After 12 h of incubation, however, the viability of the un-supplemented cultures declined significantly. In contrast, viable cell numbers were sustained or slightly increased in the glucose-supplemented cultures (Supplementary Figure S2), hence, suggesting glucose might support extended viability and stationary survival of these ED-positive isolates, at least up to 24 h.

**FIGURE 5:**
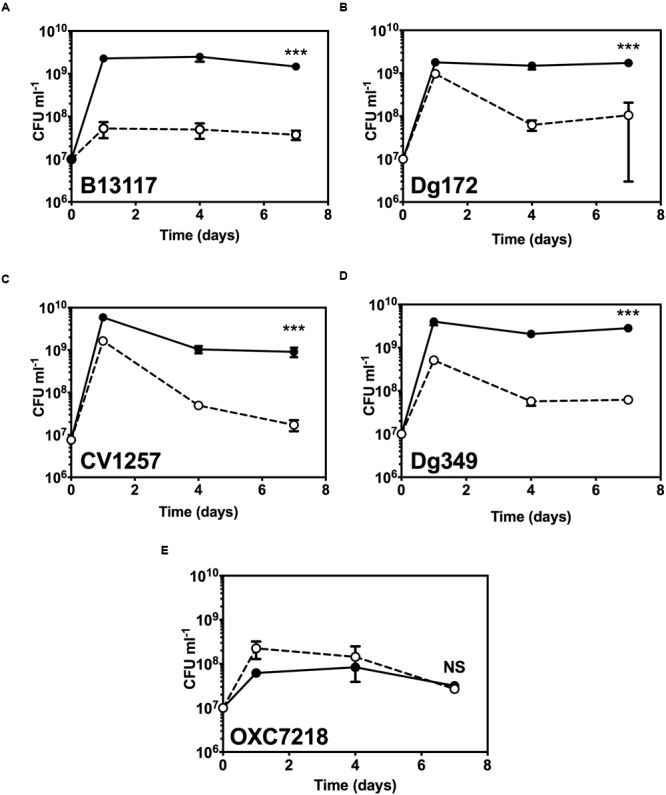
**Extended stationary phase survival with glucose.** Viable cell numbers of ED-positive *C. coli* isolates (B13117, CV1257, Dg172, and Dg349) and an ED-negative control *C. coli* strain (OXC7218) were determined in TSB with or without supplementation with 100 mM glucose. Cultures were incubated for 7 days at 37°C in microaerobic conditions and the viability determined by enumerating CFU at various time intervals. Results are mean and standard deviations of three independent cultures. The error bars are too small to be seen in some cases. **(A)** *C. coli* B13117; **(B)** *C. coli* CV1257; **(C)** *C. coli* Dg172; **(D)** *C. coli* Dg349; **(E)** *C. coli* OXC7218 (ED-negative control). Open circles and dashed line, cell numbers without glucose; filled circles and solid line, cell numbers with glucose. The statistical significance of the difference between the control without glucose and with added glucose at 7 days was tested by Student’s *t*-test, as indicated by (^∗∗∗^*p* < 0.001) or NS (not significant).

Extending the growth experiments with prolonged incubation time over several days and including four ED-positive *C. coli* isolates (B13117, CV1257, Dg172, and Dg349) and an ED-negative control *C. coli* strain (OXC7218; PubMLST id 22709), revealed that glucose did indeed support significant extended survival of the ED-positive isolates (**Figure 5**). When cultivated with glucose at 37°C in a microaerobic atmosphere, the ED-positive isolates B13117, CV1257, Dg172, and Dg349 displayed at least 10 times higher viable cell numbers after 4 and 7 days as compared to cultures without glucose, while no such effect of glucose could be seen with the ED-negative strain OXC7218 (**Figure 5**). These data show a major impact of glucose on the late stationary phase survival of ED-positive *C. coli* isolates under otherwise nutrient rich conditions.

**FIGURE 6:**
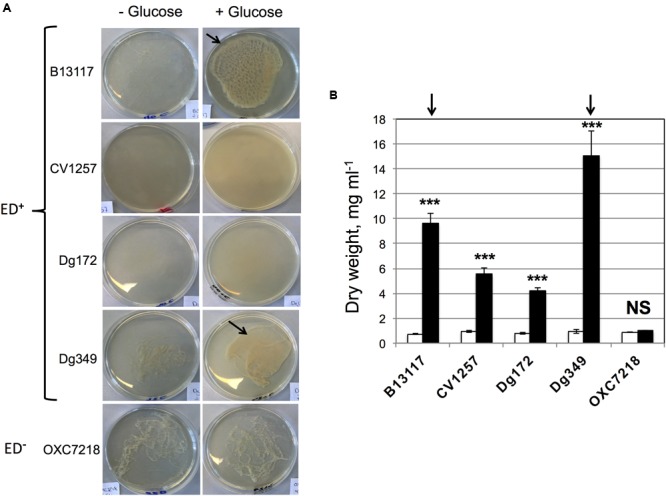
**Glucose stimulates production of floating biofilm that increases cell biomass. (A)** The isolates of *C. coli* shown were cultivated for 4 days at 37°C under microaerobic conditions in glucose-free TSB with and without supplementation with 100 mM glucose. The glucose-supplemented cultures of B13117 (ED+) and Dg349 (ED+) formed extensive floating biofilm (flocs; *arrowed*). CV1257 (ED+) and Dg172 (ED+) displayed denser growth with glucose but no biofilm formation, while a low level of aggregation was seen in the cultures of ED-negative OXC7218 regardless of glucose-supplementation. Pictures are representative cultures from four independent growths. **(B)** The four ED-positive *C. coli* isolates (B13117, CV1257, Dg172, and Dg349) and the ED-negative *C. coli* strain OXC7218 were cultivated for 7 days at 37°C under microaerobic conditions in glucose-free TSB with or without glucose supplementation. The bars show dry weight biomass of 5 ml aliquots from cultures without glucose (white bars) and with glucose supplementation (black bars). The mean and standard deviations of four independent cultures for each condition are shown. The statistical significance of the difference between the dry weight without glucose and with added glucose was evaluated by Student’s *t*-test, as indicated by (^∗∗∗^*p* < 0.001) or NS (not significant). The arrows refer to the biofilm producing strains B13117 and Dg349 as in **(A)**, which clearly produce more biomass with glucose compared to the other non-biofilm producing strains tested.

In the extended incubation experiments, it was observed that glucose stimulated biofilm formation by some ED-positive isolates of *C. coli*. Substantial floating biofilm (flocs or a pellicle), i.e., biofilm unattached to a surface, was observed in the glucose-supplemented late stationary cultures of B13117 and Dg349, and this aggregation was in fact so viscous that it was hardly dispersible by simple pipetting (**Figure 6A**). However, extensive biofilm formation was not observed in either the glucose-free cultures of B13117 and Dg349 or any of the late stationary cultures of the two other ED-positive isolates CV1257 and Dg172 in the presence of glucose. The ED-negative strain OXC7218 displayed a low level of aggregation regardless of the glucose availability (**Figure 6A**). Note that the biofilm produced by *C. coli* B13117 and Dg349 is floating, i.e., unattached to a solid surface, and therefore it was not possible to quantify using traditional dye staining assays for staining of attached biofilms. Consequently, the contribution of the biofilm to the total cell biomass was quantified using the dry weight of culture aliquots following 7 days of incubation with and without glucose (**Figure 6B**). As expected, this showed a significant increase in the biomass of all the ED-positive isolates upon glucose supplementation, while the biomass of the ED-negative strain was unaltered regardless of glucose availability. However, the biomass of B13117 and Dg349 grown with glucose was clearly much greater than that of CV1257 and Dg172 with glucose (**Figure 6B**, arrows), which is in agreement with the visually observed high biofilm production by these isolates (**Figure 6A**, arrows).

### Exometabolic Profiling of ED-Positive Isolates Grown with and without Glucose

**FIGURE 7:**
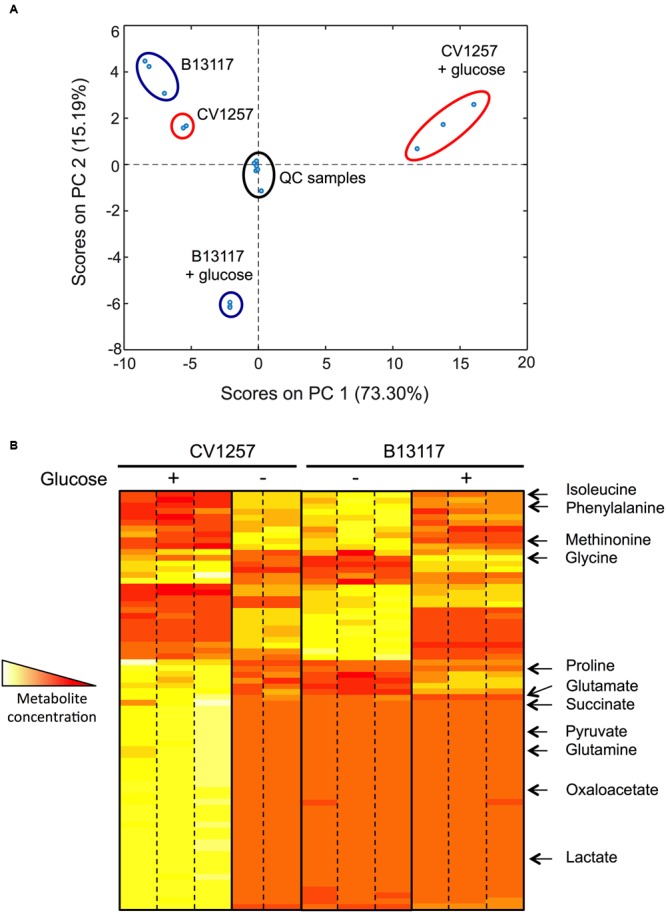
**Glucose dependent changes in extracellular metabolic profiles of B13117 and CV1257 isolates. (A)** Principal components analysis (PCA) plots of extracellular metabolites determined by gas chromatography–mass spectrometry (GC–MS). The PCA score plot is based on a data set consisting of three independent replicates of *C. coli* B13117 and CV1257 cultivated in TSB with and without glucose for 24 h at 37°C in a microaerobic atmosphere. However, only two replicates of CV1257 without glucose were used due to contamination. For quality control, mixed pooled samples (labeled QC samples) were run alongside the individual separate samples. **(B)** Heat map representation of the most abundant extracellular metabolites of *C. coli* B13117 and CV1257 samples used in **(A)**. When cultivated without glucose the two isolates display very similar metabolite profiles (two middle panels). Upon glucose supplementation both isolates display a distinct shift in metabolic profile (two outer panels), but the two isolates respond very differently to glucose metabolism, which is seen by the highly different overall profile patterns with glucose. Compounds that could be identified by comparison to standards are shown; most compounds that could be detected were not identified. The colors represent the relative concentrations from high (white) to low (red).

*Campylobacter coli* B13117 and CV1257 were subjected to an exometabolomics “footprint” analysis to compare the extracellular metabolic profiles of these ED-positive isolates while growing in TSB with and without glucose. Changes in the media composition after growth, reflecting the metabolic activity of the bacteria (i.e., production and/or consumption of metabolites and changes in growth substrates) were evaluated using a validated method based on gas chromatography–mass spectrometry (GC–MS) analysis of methyl chloroformate derivatised samples (Smart et al., 2010) which primarily identifies amino-acids and organic acids. In the principal component analysis (PCA) shown in **Figure 7A**, it is clear that the footprints of B13117 and CV1257 are highly similar when the isolates are cultivated in TSB without glucose, as these samples group closely together in the same region of the PCA plot (**Figure 6A**). However, upon glucose supplementation, the samples from the two isolates group in opposite quadrants of the plot (**Figure 6A**), indicating a substantial but very dissimilar response to glucose. A heat map, representing the relative concentrations of the compounds detected by GC–MS, is shown in **Figure 7B**. The largely similar extracellular metabolic profiles of B13117 and CV1257, when cultivated in TSB without glucose, are clear from the overall pattern seen in the heat map profile. After growth with glucose a clear difference between the two isolates in their responses in terms of metabolite concentrations was observed (**Figure 7B**). Using known reference standards, some of these extracellular compounds could be identified. From the results, it seems that glucose stimulates catabolism more strongly in CV1257, since the extracellular concentration of several metabolites both from and closely related to the citric-acid cycle (e.g., succinate, pyruvate, oxaloacetate, lactate) was found to increase when this strain was cultivated with glucose as compared to a glucose-free medium, while the amino-acids isoleucine, phenylalanine, and methionine decreased (**Figure 7B**). The metabolic profile of B13117 also displayed a clear shift upon cultivation with glucose, with certain amino-acids from the growth medium again decreasing. However, far fewer glucose-dependent extracellular metabolite alterations of B13117 were identified with the GC–MS analysis, and in particular there was little change in citric-acid cycle related organic acids (**Figure 7B**). Given the strong tendency of the B13117 strain, but not CV1257, to produce biofilm upon cultivation with glucose (**Figure 6**), the distinct metabolic profile compared to CV1257 is consistent with glucose carbon being preferentially directed into polysaccharide formation in the former isolate.

### Genomic Comparison of B13117 and CV1257

In addition to metabolic differences in the B13117 and CV1257 isolates, we also searched for possible differences in gene content that might be related to polysaccharide formation, in particular in the capsule biosynthesis loci. A total of 1,996 unique coding sequences were identified between B13117 and CV1257, 1,618 of which were present in both isolates. Among the remaining coding sequences, 204 and 174 were specific to B13117 and CV1257, respectively. Differences in the capsule biosynthesis loci accounted for 35 (9.3%) of the isolate-specific genes, with the region being approximately 5.6 kb longer in B13117 (Supplementary Figure S3). Although the flanking genes shared >90% sequence identity, the intervening sequences were distinct (Supplementary Figure S3). Some of the additional genes in this region in B13117 have predicted functions in polysaccharide biosynthesis (Supplementary Table S3). With respect to the remaining isolate-specific genes, 174 corresponded to hypothetical proteins.

## Discussion

Glucose utilization by *Campylobacter* strains has only recently been discovered, and it remains of uncertain physiological, pathogenic and environmental significance. Taken together, the results of this study illustrate that while the ED pathway is indeed present in diverse isolates in the *Campylobacter* genus, overall it is uncommon, as judged by the presence of the complete *glc* locus in only 1.7% of over 6,000 genomes analyzed. Importantly, we did not find a correlation between possession of the key ED pathway genes and the ability to cause bloodstream infections, which might have been expected given the abundance of glucose in this niche. However, from our genome sequence analyses, it is clear that the ED pathway is present in both *C. jejuni* and *C. coli* isolates and we believe that this is the first report of this pathway in *C. jejuni* subsp. *jejuni*. The previous study of Vorwerk et al. (2015) employed a limited number of human disease and pig *C. coli* isolates, while we have shown that the *glc* locus is clearly conserved among *C. coli* from more diverse sources. The majority of genomes in the *C. jejuni*/*coli* PubMLST database were from human disease cases, and, as humans typically become infected due to consumption of contaminated animal products (Kaakoush et al., 2015), the isolates analyzed effectively comprised an agricultural sample. This raises questions about the advantages to *C. coli* of glucose utilization in agricultural hosts. It is known that the *glc* locus forms a plasticity region that can be transferred by natural transformation between isolates (Vorwerk et al., 2015), so it is likely that the region has been gained or lost repeatedly in different isolates possibly in response to the differing availability of glucose within distinct hosts or niches.

The genes comprising the *glc* locus were most variable among genetically diverse *C. jejuni* isolates that were primarily not from agricultural sources, but were from rats and wild birds and also corresponded to *C. jejuni* subsp. *doylei*, the natural reservoir of which remains unknown. Identification of clusters of closely related *C. jejuni* from rats that shared identical ED types was likely due to sampling: isolates with ED type 2 were recovered primarily from two different farms over 18 months, while four of the five isolates with ED type 3 were recovered from a third farm over the same period (MacIntyre, in preparation). In contrast, although *C. coli* isolates were also genetically diverse at the wgMLST level, the *glc* locus was less variable in these isolates, 73% of which corresponded to ED type 1. This difference in *glc* diversity between *C. jejuni* and *C. coli* may be related to when the region was gained or lost with respect to the timing of divergence of *Campylobacter* species/subspecies, or to host differences, or to sparse sampling of *C. coli* from non-agricultural sources. Whether this has any physiological consequences in terms of overall pathway activity or specific enzyme activity will require further investigation. There were also slight differences in the gene contents of the *glc* locus, with wild bird isolates carrying an additional ∼2700 bp downstream of *glcP*. Further work is needed to understand the significance of this with respect to the function of the pathway as a whole.

We have shown for the first time that individual glucose metabolizing isolates can show distinct physiological responses to the availability of this substrate, which suggests that glucose can act as more than just an energy source for campylobacters. What are the additional specific physiological advantages of the possession of the ED pathway by a subset of *Campylobacter* isolates? The production of NADPH by glucose-6-phosphate dehydrogenase may be particularly significant, as this is not only required for anabolic enzyme reactions, but also supplies reductant for oxidative stress protection, mainly via the thioredoxin system (Lu and Holmgren, 2014). In the oxygen-sensitive microaerophilic campylobacters, this system (NADPH + thioredoxin reductase + thioredoxin) is crucial, as it supplies electrons to the peroxiredoxins AhpC, Tpx and Bcp, each of which has been shown to protect against oxidative stress by removing damaging peroxides (Baillon et al., 1999; Atack et al., 2008). Thioredoxin also reduces the cytoplasmic methionine sulfoxide reductases MsrA and MsrB, which repair oxidized proteins (Atack and Kelly, 2008). Thus, ED-positive isolates may be better able to cope with oxidative stress than ED-negative isolates; this has been shown to be the case experimentally in *Pseudomonas putida* (Chavarria et al., 2013) and in a range of marine bacteria (Klingner et al., 2015). Secondly, the absence of Pfk but the presence of the remaining EMP pathway enzymes in campylobacters means that the ED reactions are not “linear.” Instead, the metabolism of glucose in ED-positive isolates could occur via the “cyclic” fusion of the ED and EMP reactions, as has been shown in *Pseudomonas* spp., which interestingly also lack Pfk (Nikel et al., 2015). As suggested for pseudomonads, the physiological rationale for this might be related to an enhanced hexose phosphate supply; this would be particularly important for LOS, capsule and polysaccharide formation in campylobacters.

In the Biolog^TM^ assays and liquid batch culture growth experiments in minimal media, we confirmed that glucose utilization and glucose-stimulated growth, respectively, occurred in several ED-positive but not ED-negative isolates. This was as expected and is in agreement with the findings of Vorwerk et al. (2015). However, we found that addition of glucose to the ED-positive isolates in rich, complex media, afforded a significant stationary phase survival benefit, in terms of the maintenance of viability over several days compared to the absence of glucose. This effect is likely a product of the multiple functions of the ED pathway in energy conservation, NADPH production and hexose phosphate generation, which may allow better environmental resilience. Moreover, in further physiological studies on glucose utilization in rich complex media, we found some unexpected differences between the ED-positive isolates with regard to biofilm formation. It was clear from the dry weight measurements and visual appearance of the cultures that glucose utilization led to a massive increase in a pellicle or floating biofilm in isolates like B13117 and Dg349 but not CV1257 and Dg172, which suggested a fundamental difference in the way in which glucose is being metabolized in these isolates. This conclusion was supported by the metabolic profiling analysis we carried out using GC–MS of broth culture supernatants, where we could distinguish a distinct metabolic footprint when comparing the high biofilm forming B13117 strain with the low biofilm forming CV1257. In the latter strain only, several citric-acid cycle organic acids and also lactate were increased in the culture supernatants in a glucose dependent manner. Our conclusion is therefore that the ED pathway in CV1257 primarily feeds glucose into the primary metabolism of this strain. In contrast, B13117 may primarily utilize the ED encoded glucose kinase (Glk) and possibly the EMP gluconeogenic reactions, for conversion of glucose to glucose-6-phosphate and/or fructose-6-phosphate which could be used for the production of surface structures such as capsule or free polysaccharide. This would be consistent with the observed massive increase in biofilm production by this strain when cultivated with glucose.

Although, future analysis of gene expression patterns and *in vivo* activities of ED and EMP pathway enzymes will be required to explain the molecular basis for the different metabolic responses to glucose in B13117 and CV1257, we found differences in gene content in these isolates that might also contribute to the differences in biofilm formation, with 378 isolate-specific coding sequences identified. We speculate that some of these might be involved in polysaccharide synthesis specifically related to biofilm formation. For example, the capsule biosynthesis locus is ∼5.6 kB longer in B13117, and, although the flanking regions are conserved, the intervening sequences are distinct (Supplementary Figure S3). For example, there are several distinct *rbf* genes in B13117: glucose-1-phosphate cytidylyltransferase (*rfbF*), CDP-glucose 4,6-dehydratase (*rfbG*), and CDP-abequose synthase (*rbfJ*), that encode enzymes of polysaccharide synthesis, which might contribute to the observed biofilm formation (Supplementary Table S3). The role of these and other polysaccharide biosynthesis genes, biochemical analysis of the biofilm polymer together with further detailed analysis of gene function and regulation and enzyme activities will elucidate the different phenotypes of these isolates seen in the presence of glucose. It would also be informative to determine how common the glucose stimulated survival and biofilm phenotypes are by screening a much larger number of isolates than was possible in this study.

Finally, there is overwhelming evidence showing that biofilm production is of prime importance in many aspects of the biology of *Campylobacter* isolates, particularly protection against environmental stress (e.g., Svensson et al., 2009; Haddock et al., 2010; Bae and Jeon, 2013; Pascoe et al., 2015) and that it is affected by multiple external factors such as medium composition, osmolarity and oxygen availability (Reeser et al., 2007; Reuter et al., 2010). Host signals also play a role; a recent study showed that *C. jejuni* 11168 can produce a glucan biofilm composed of α-dextran as a specific response to the presence of host pancreatic amylase (Jowiya et al., 2015). In contrast to the clear stimulatory effect of glucose on biofilm formation we found here, the only other sugar known to be metabolized by campylobacters, L-fucose, was very recently shown to *reduce* biofilm formation in the *C. jejuni* NCTC 11168 strain (Dwivedi et al., 2016). The reduction was dependent on L-fucose transport and metabolism and the authors speculated that L-fucose might be an intestinal signal to maintain cells in a planktonic state. Taking our data together with the findings of Dwivedi et al. (2016) it is now clear that these two related hexose sugars are not only metabolized very differently, but they also play very different roles in modulating the crucial biofilm response of campylobacters.

## Author Contributions

CV, JR, LJ, MD, designed and executed experiments and analyzed the data. MJ performed the sequencing and bioinformatics analyses. CV and DK wrote the manuscript. MJ, MM, SM, HI, LJ, MD, and DK critically evaluated and revised the manuscript.

## Conflict of Interest Statement

The authors declare that the research was conducted in the absence of any commercial or financial relationships that could be construed as a potential conflict of interest.
