# Supplementary material for: Glucose Metabolism via the Entner-Doudoroff Pathway in Campylobacter: A Rare Trait that Enhances Survival and Promotes Biofilm Formation in Some Isolates
Source: Front Microbiol. 2016 Nov 22;7:1877. doi: 10.3389/fmicb.2016.01877 (PMC5118423; doi:10.3389/fmicb.2016.01877)
Supplement: Supplementary file 1 [file Presentation_1.PDF]

*Supplementary Material*

# Glucose metabolism via the Entner-Doudoroff pathway in *Campylobacter*: A rare trait that enhances survival and promotes biofilm formation in some isolates

Christina S. Vegge, Melissa J. Jansen van Rensburg, Janus J. Rasmussen, Martin C.J. Maiden, Lea G. Johnsen, Morten Danielsen, Sheila MacIntyre, Hanne Ingmer and David J. Kelly [*]

[*] Correspondence: Professor David J. Kelly: d.kelly@sheffield.ac.uk

**Supplementary Table 1. *Campylobacter* strains used experimentally in this study**

| Species | Isolate | Origin | ED | Reference |
|---|---|---|---|---|
| *C. jejuni* subsp. *doylei* | 269.97 | 1977, South Africa, human blood | + | Fouts *et al.*, 2007. Direct submission |
| | CCUG18265 | 1984, Germany, gastric biopsy | + | Culture collection, University of Göteborg |
| | CCUG24567 | 1986, Australia, Infantile diarrhea | + | Culture collection, University of Göteborg |
| | CCUG26152 | 1990, Belgium, human feces, child | - | Culture collection, University of Göteborg |
| | CCUG26155 | 1990, Belgium, human feces, child | + | Culture collection, University of Göteborg |
| | CCUG36506/ NCTC 11951 | Australia, human Feces, 2-year-old child | - | Culture collection, University of Göteborg. Steele and Owen, 1988 |
| *C. jejuni* subsp. *jejuni* | NCTC11168 | 1977, feces of a diarrheic patient | - | Parkhill *et al.*, 2000 |
| | S38543 | Denmark, bacteremia | - | Statens Serum Institute, Denmark |
| | B10540 | Denmark, bacteremia | - | J. Engberg, Hovedstadens Sygehusvæsen, Denmark |
| | B345 | Denmark, bacteremia | - | J. Engberg, Hovedstadens Sygehusvæsen, Denmark |
| | B15426 | Denmark, bacteremia | - | J. Engberg, Hovedstadens Sygehusvæsen, Denmark |
| | B130 | Denmark, bacteremia | - | J. Engberg, Hovedstadens Sygehusvæsen, Denmark |
| | B17648 | Denmark, bacteremia | - | J. Engberg, Hovedstadens Sygehusvæsen, Denmark |
| | B19978 | Denmark, bacteremia | - | J. Engberg, Hovedstadens Sygehusvæsen, Denmark |
| | B3-11 | Denmark, bacteremia | - | J. Engberg, Hovedstadens Sygehusvæsen, Denmark |
| | B5682 | Denmark, bacteremia | - | J. Engberg, Hovedstadens Sygehusvæsen, Denmark |
| | B10053 | Denmark, bacteremia | - | J. Engberg, Hovedstadens Sygehusvæsen, Denmark |
| *C. coli* | B13117 | 2011, Denmark, bacteremia | + | J. Engberg, Hovedstadens Sygehusvæsen, Denmark |
| | CV1257 | Unknown | + | K. Jørgensen, University of Copenhagen |
| | OXC6725 | 2011, UK, gastroenteritis, human stool | - | Oxford University |
| | OXC7218 | 2012, UK, gastroenteritis, human stool | - | Oxford University |
| | Dg172 | 2012, UK, pig farm associated Norway rat | + | S. McIntyre, University of Reading |
| | Dg349 | 2012, UK, sheep farm associated Norway rat | + | S. McIntyre, University of Reading |

**Supplementary Table 2. PubMLST identifiers and European Nucleotide Archive accession numbers for the 113 *Campylobacter* isolates with complete *glc* loci analysed in this study**

| Isolate name | PubMLST id | ENA accession |
|---|---|---|
| 269.97/RM4099[a] | 4312 | NC_009707 |
| OXC6559 | 12894 | ERR108323 |
| OXC6744 | 18301 | ERR137005 |
| OXC7027 | 22214 | ERR221311 |
| OXC7051 | 22236 | ERR221333 |
| Dg356 | 24192 | ERR278293 |
| 8993 | 24255 | |
| OXC7612 | 24541 | ERR330519 |
| OXC7653 | 24908 | ERR343078 |
| Dg20a | 25937 | ERR348962 |
| Dg217 | 25945 | ERR348984 |
| Dg57a | 25947 | ERR348986 |
| Dg268 | 25948 | ERR348992 |
| Dg44a | 25950 | ERR348994 |
| Dg26b | 25959 | ERR349015 |
| Dg69 | 25962 | ERR349018 |
| Dg22 | 25974 | ERR349042 |
| Dg63a | 25975 | ERR349047 |
| Dg43a | 25978 | ERR349050 |
| Dg349[a] | 25986 | ERR349061 |
| Dg80a | 25987 | ERR349062 |
| Dg131 | 25988 | ERR349063 |
| Dg224 | 25989 | ERR349064 |
| Dg307 | 25990 | ERR349065 |
| Dg304 | 25991 | ERR349066 |
| Dg81 | 25995 | ERR349070 |
| Dg233 | 25997 | ERR349072 |
| Dg122 | 25999 | ERR349074 |
| Dg86 | 26004 | ERR349079 |
| Dg128 | 26007 | ERR349082 |
| Dg345 | 26009 | ERR349084 |
| Dg130 | 26015 | ERR349090 |
| Dg109 | 26019 | ERR349094 |
| Dg95 | 26020 | ERR349095 |
| Dg294 | 26022 | ERR349097 |
| Dg210 | 26027 | ERR349102 |
| Dg137 | 26031 | ERR349106 |
| Dg180 | 26034 | ERR349109 |
| Dg184 | 26040 | ERR349115 |
| Dg104 | 26043 | ERR349119 |
| Dg277 | 26045 | ERR349121 |
| Dg234 | 26049 | ERR349126 |
| Dg187 | 26054 | ERR349131 |

| | | |
|---|---|---|
| Dg275 | 26058 | ERR349136 |
| Dg246 | 26063 | ERR349142 |
| Dg117 | 26064 | ERR349143 |
| Dg172[a] | 26067 | ERR349146 |
| OXC8567 | 28906 | ERR585790 |
| H143980374 | 31007 | |
| CCUG 24567[a] | 31098 | |
| CCUG 18265[a] | 31099 | |
| CCUG 26155[a] | 31100 | |
| B13117[a] | 31101 | |
| H145160764 | 32502 | |
| H151180505 | 32710 | |
| H151840727 | 33283 | |
| H151840499 | 33285 | |
| Uoh_CB287 | 33931 | |
| Uoh_CB291 | 33934 | |
| Uoh_CB292 | 33935 | |
| Uoh_CB300 | 33939 | |
| Uoh_CB301 | 33940 | |
| Uoh_CB303 | 33941 | |
| Uoh_CB304 | 33942 | |
| Uoh_CB305 | 33943 | |
| Uoh_CB306 | 33944 | |
| Uoh_CB307 | 33945 | |
| Uoh_CB309 | 33947 | |
| Uoh_CB311 | 33948 | |
| Uoh_CB313 | 33949 | |
| Uoh_CB316 | 33950 | |
| Uoh_CB318 | 33951 | |
| Uoh_CB319 | 33952 | |
| Uoh_CB334 | 33957 | |
| Uoh_CB335 | 33958 | |
| Uoh_CB340 | 33959 | |
| Uoh_CB342 | 33960 | |
| Uoh_CB344 | 33961 | |
| Uoh_CB356 | 33967 | |
| Uoh_CB357 | 33968 | |
| H152360340 | 33981 | |
| H152360660 | 33982 | |
| Uoh_CB372 | 34070 | |
| Uoh_CB379 | 34073 | |
| Uoh_CB383 | 34076 | |
| Uoh_CB386 | 34078 | |
| Uoh_CB387 | 34079 | |
| Uoh_CB388 | 34080 | |
| Uoh_CB389 | 34081 | |
| Cc67-8 | 35271 | |

| | | |
|---|---|---|
| OXC10074 | 43772 | |
| FS101087_LDI12898 | 43942 | ERR1163392 |
| FS101087_LDI12914 | 43958 | ERR1163408 |
| FS101087_LDI13539 | 44027 | ERR1163477 |
| FS101087_LDI4910 | 44161 | ERR1163610 |
| FS101087_LDI6793 | 44223 | ERR1163672 |
| FS101087_LDI6798 | 44228 | ERR1163677 |
| FS101087_LDI6806 | 44236 | ERR1163685 |
| FS101087_LDI9133 | 44275 | ERR1163724 |
| FS101087_LDI9139 | 44281 | ERR1163730 |
| FS101087_LDI9145 | 44287 | ERR1163736 |
| FS101087_LDI9156 | 44298 | ERR1163747 |
| FS101087_LDI9164 | 44306 | ERR1163755 |
| FS101087_LDI9192 | 44333 | ERR1163782 |
| FS101087_LDI9878 | 44359 | ERR1163808 |
| FS101087_LDI9880 | 44361 | ERR1163810 |
| FS101087_LDI9883 | 44364 | ERR1163813 |
| FS101087_LDI9899 | 44380 | ERR1163829 |
| FS101087_LDI9902 | 44383 | ERR1163832 |
| FS101087_LDI9914 | 44394 | ERR1163843 |
| FS101087_LDI9931 | 44411 | ERR1163860 |
| FS101087_LDI9933 | 44413 | ERR1163862 |
| CV1257[a] | 48095 | |

[a] Isolates included experiments

**Supplementary Table 3. Gene predictions in the capsule biosynthesis locus of B13117 and CV1257**.

| Prokka locus | Predicted gene name (if available) | Predicted product |
|---|---|---|
| B13117cps_00001 | kpsM | Polysialic acid transport protein KpsM |
| B13117cps_00002 | kpsT | Polysialic acid transport ATP-binding protein KpsT |
| B13117cps_00003 | | hypothetical protein |
| B13117cps_00004 | kpsD | Polysialic acid transport protein KpsD precursor |
| B13117cps_00005 | kdsD | Arabinose 5-phosphate isomerase KdsD |
| B13117cps_00006 | | Oxidoreductase family%2C NAD-binding Rossmann fold |
| B13117cps_00007 | | hypothetical protein |
| B13117cps_00008 | moaA | Cyclic pyranopterin monophosphate synthase |
| B13117cps_00009 | epsN | Putative pyridoxal phosphate-dependent aminotransferase EpsN |
| B13117cps_00010 | epsM | Putative acetyltransferase EpsM |
| B13117cps_00011 | legI | N%2CN'-diacetyllegionaminic acid synthase |
| B13117cps_00012 | carB | Carbamoyl-phosphate synthase large chain |
| B13117cps_00013 | gph | Phosphoglycolate phosphatase |
| B13117cps_00014 | legG | GDP/UDP-N%2CN'-diacetylbacillosamine 2-epimerase (hydrolyzing) |
| B13117cps_00015 | | GlcNAc-PI de-N-acetylase |
| B13117cps_00016 | hddC | D-glycero-alpha-D-manno-heptose 1-phosphate guanylyltransferase |
| B13117cps_00017 | neuA | CMP-N%2CN'-diacetyllegionaminic acid synthase |
| B13117cps_00018 | | Glycosyl transferase family 8 |
| B13117cps_00019 | | hypothetical protein |
| B13117cps_00020 | | Glycosyl transferase family 2 |
| B13117cps_00021 | rfbF | Glucose-1-phosphate cytidylyltransferase |
| B13117cps_00022 | btrR | L-glutamine:2-deoxy-scyllo-inosose aminotransferase |
| B13117cps_00023 | rfbG | CDP-glucose 4%2C6-dehydratase |
| B13117cps_00024 | rmlC | dTDP-4-dehydrorhamnose 3%2C5-epimerase |
| B13117cps_00025 | rfbJ | CDP-abequose synthase |
| B13117cps_00026 | mnmC | tRNA 5-methylaminomethyl-2-thiouridine biosynthesis bifunctional protein MnmC |
| B13117cps_00027 | | hypothetical protein |
| B13117cps_00028 | | Capsule polysaccharide biosynthesis protein |
| B13117cps_00029 | | Capsule polysaccharide biosynthesis protein |
| CV1257cps_00001 | | Capsule polysaccharide biosynthesis protein |
| CV1257cps_00002 | | Capsule polysaccharide biosynthesis protein |
| CV1257cps_00003 | | hypothetical protein |
| CV1257cps_00004 | arnB | UDP-4-amino-4-deoxy-L-arabinose--oxoglutarate aminotransferase |
| CV1257cps_00005 | fcl | GDP-L-fucose synthase |
| CV1257cps_00006 | rfbC | dTDP-4-dehydrorhamnose 3%2C5-epimerase |
| CV1257cps_00007 | gmd | GDP-mannose 4%2C6-dehydratase |
| CV1257cps_00008 | algA | Alginate biosynthesis protein AlgA |
| CV1257cps_00009 | fcf2 | dTDP-fucopyranose mutase |
| CV1257cps_00010 | | F5/8 type C domain protein |
| CV1257cps_00011 | epsJ | putative glycosyltransferase EpsJ |
| CV1257cps_00012 | | hypothetical protein |
| CV1257cps_00013 | | hypothetical protein |
| CV1257cps_00014 | | CDP-Glycerol:Poly(glycerophosphate) glycerophosphotransferase |
| CV1257cps_00015 | ispD2 | Putative 2-C-methyl-D-erythritol 4-phosphate cytidylyltransferase 2 |
| CV1257cps_00016 | kdsD | Arabinose 5-phosphate isomerase KdsD |
| CV1257cps_00017 | kpsD | Polysialic acid transport protein KpsD precursor |
| CV1257cps_00018 | | hypothetical protein |
| CV1257cps_00019 | kpsT | Polysialic acid transport ATP-binding protein KpsT |
| CV1257cps_00020 | kpsM | Polysialic acid transport protein KpsM |

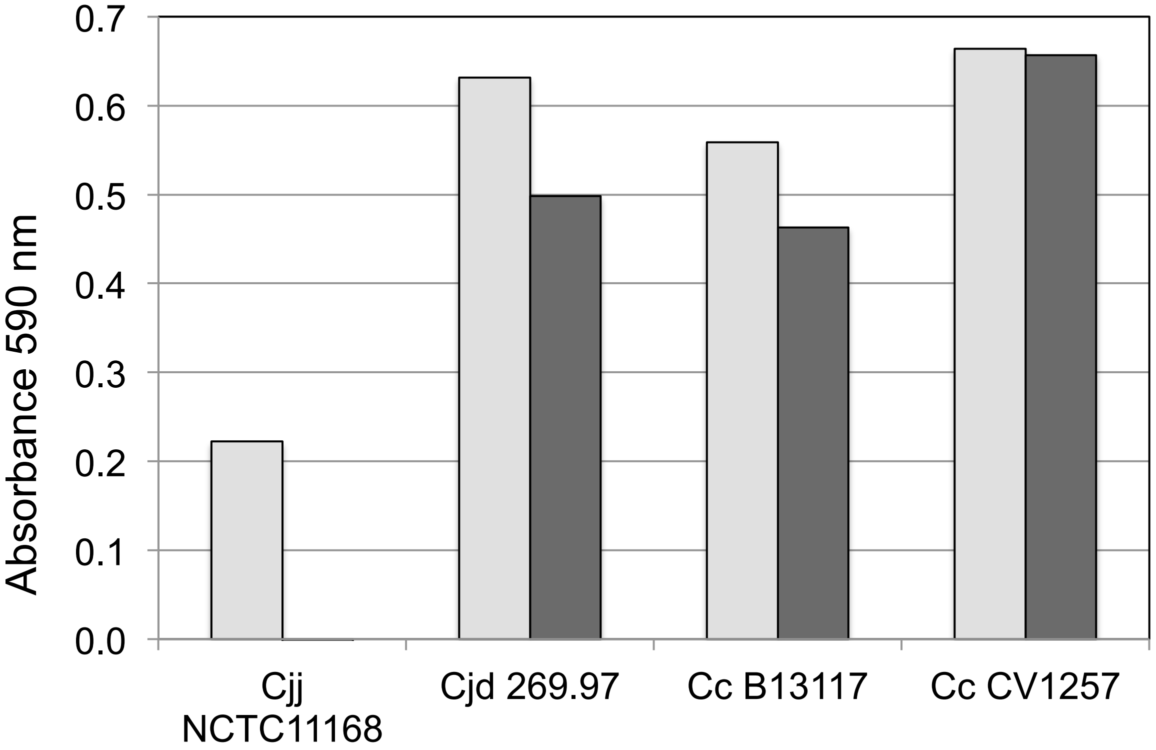


**Supplementary Figure 1. Glucose stimulated respiration of ED-positive *Campylobacter* isolates.** Results from Biolog phenotypic microarray plate PM1 after 24 h incubation at 37 °C under microaerobic conditions. Light grey bars; stimulation of respiration from uptake and metabolism of L-fucose. Dark grey bars; stimulation of respiration from uptake and metabolism of D-glucose. *C. jejuni* subsp. *doylei* 269.97, *C. coli* B13117 and *C. coli* CV1257 encode the ED pathway, while *C. jejuni* subsp. *jejuni* NCTC11168 does not. Presented results are from one experiment, corrected for background absorbance. An independent replicate showed the same glucose stimulated respiration in the Cjd and Cc strains but not NCTC11168.

A

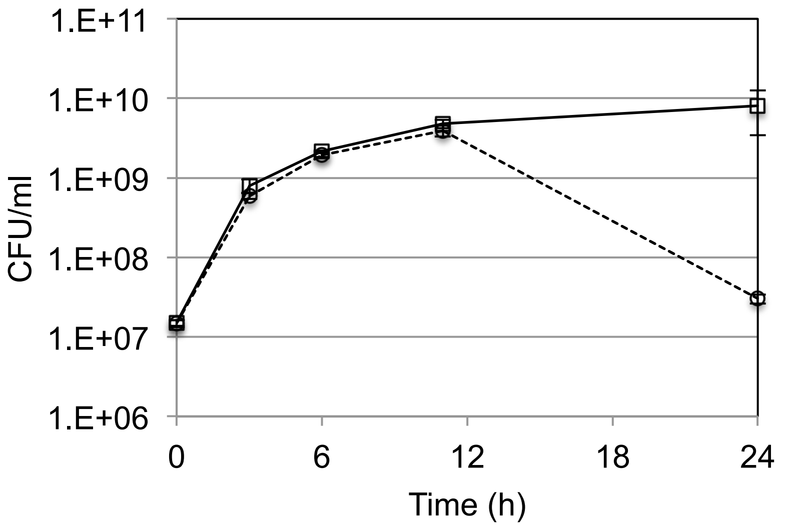


B

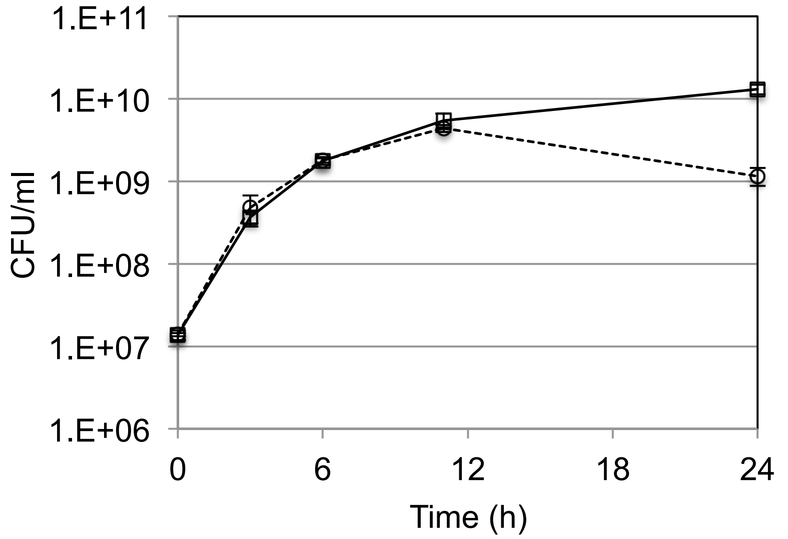


**Supplementary Figure 2. Growth patterns of two ED-positive *C. coli* isolates in rich media with or without glucose.** *C. coli* B13117 (A) and CV1257 (B) were inoculated into both glucose-free TSB (dashed lines) and TSB supplemented with 100 mM glucose (solid lines). Cultures were incubated at 37 °C under microaerobic conditions and the viable cell numbers determined by enumerating CFU at various time intervals. Results shown are mean and standard deviations of three independent cultures for each condition.

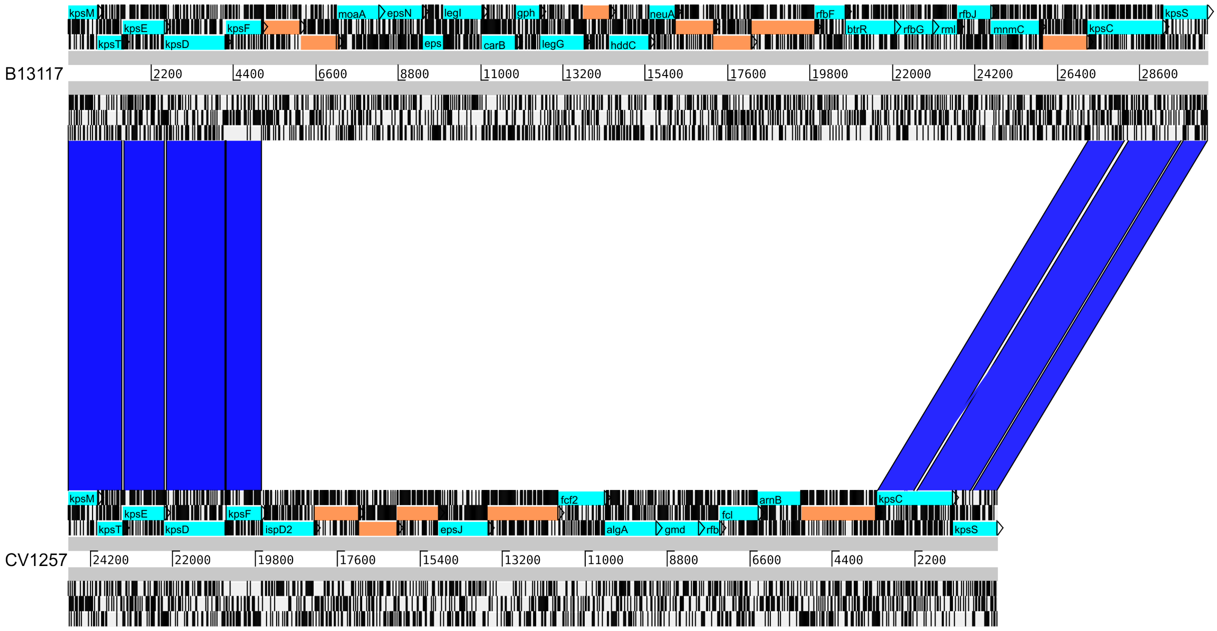


**Supplementary Figure 3. Comparison of capsule biosynthesis gene region in isolates B13117 and CV1257.** The genomes were annotated using Prokka, the *kpsM* and *kpsS* genes identified, and these genes and the intervening regions extracted and compared using the Artemis Comparison Tool (ACT). Blue bars between the sequences mark regions with a sequence identity >70%. Coding sequences of hypothetical proteins are shown in orange.
